# Supplementary material for: Metabolomic signatures associated with fetal growth restriction and small for gestational age: a systematic review
Source: Nat Commun. 2024 Nov 11;15:9752. doi: 10.1038/s41467-024-53597-4 (PMC11555221; doi:10.1038/s41467-024-53597-4)
Supplement: Supplementary file 1 — Supplementary Information [file 41467_2024_53597_MOESM1_ESM.pdf]

SUPPLEMENTARY INFORMATION

Metabolomic signatures associated with fetal growth restriction and small for gestational age: a systematic review

Agustin Conde-Agudelo[1]*, Jose Villar[1,2]*, Milagros Risso[3], Aris T. Papageorghiou[1,2], Lee D. Roberts[4] & Stephen H. Kennedy[1,2]

1. Oxford Maternal & Perinatal Health Institute, Green Templeton College, University of Oxford, Oxford, UK
2. Nuffield Department of Women's & Reproductive Health, University of Oxford, Oxford, UK
3. Hospital Universitario General de Villalba, Madrid, Spain
4. Leeds Institute of Cardiovascular and Metabolic Medicine, University of Leeds, Leeds, UK

Corresponding authors: Agustin Conde-Agudelo condeagu@hotmail.com and Jose Villar jose.villar@wrh.ox.ac.uk

Table of Contents

Supplementary Table 1. Results from the pathway analysis in umbilical cord blood samples.

Supplementary Table 2. Results from the pathway analysis in newborn dried blood spot samples.

Supplementary Table 3. Results from the pathway analysis in maternal plasma/serum at >20 weeks' gestation or in the peripartum period.

Supplementary Table 4. Results from the pathway analysis in placental samples.

Supplementary Figure 1. Risk of bias for each included study.

Supplementary Figure 2. Pathway analysis for significantly and consistently up- and down-regulated metabolites (N=5) that were reported in more than one study in maternal plasma/serum at >20 weeks' gestation or in the peripartum period.

Supplementary Figure 3. Pathway analysis for significantly and consistently up- and down-regulated metabolites (N=3) that were reported in more than one study in placental samples.

Supplementary Box 1. Metabolites significantly up-regulated or down-regulated in more than one study with a consistent direction of the association

Supplementary Table 1 | Results from the pathway analysis in umbilical cord blood samples.

Pathway name	Match status	Raw p	Holm adjusted p	FDR p	Impact
Biosynthesis of unsaturated fatty acids	4/36	1.959E-4	0.015672	0.015672	0.0
Glycerophospholipid metabolism	3/36	0.00336	0.26544	0.1344	0.13764
Neomycin, kanamycin and gentamicin biosynthesis	1/2	0.017704	1.0	0.47212	0.0
Linoleic acid metabolism	1/5	0.043716	1.0	0.87432	0.0
Arachidonic acid metabolism	2/44	0.056108	1.0	0.89773	0.27659
Valine, leucine and isoleucine biosynthesis	1/8	0.069087	1.0	0.92115	0.0
alpha-Linolenic acid metabolism	1/13	0.10999	1.0	1.0	0.0
Starch and sucrose metabolism	1/18	0.14921	1.0	1.0	0.4207
Ether lipid metabolism	1/20	0.16445	1.0	1.0	0.08176
Galactose metabolism	1/27	0.2158	1.0	1.0	0.03499
Lysine degradation	1/30	0.2369	1.0	1.0	0.11247
Glycine, serine and threonine metabolism	1/33	0.25747	1.0	1.0	0.0
Arginine and proline metabolism	1/36	0.27753	1.0	1.0	0.02093
Valine, leucine and isoleucine degradation	1/40	0.30348	1.0	1.0	0.0
Steroid biosynthesis	1/41	0.30984	1.0	1.0	0.02837

Primary bile acid biosynthesis	1/46	0.3408	1.0	1.0	0.05065
Fatty acid biosynthesis	1/47	0.34684	1.0	1.0	0.0
Steroid hormone biosynthesis	1/87	0.55018	1.0	1.0	0.00523

The table shows the detailed results from the pathway analysis. Since many pathways are tested at the same time, the statistical p values from enrichment analysis are further adjusted for multiple testings. Match status reflects hits/total, where total means the total number of compounds in the pathway, and the hits are the actually matched compounds from the list; the Raw p is the original p value calculated from the enrichment analysis; the Holm p is the adjusted p value calculated from the Holm-Bonferroni method; the FDR p is the p value adjusted using False Discovery Rate; the Impact is the pathway impact value calculated from pathway topology analysis. *FDR* false discovery rate.

Supplementary Table 2 | Results from the pathway analysis in newborn dried blood spot samples.

Pathway name	Match status	Raw p	Holm adjusted p	FDR p	Impact
Phenylalanine, tyrosine and tryptophan biosynthesis	2/4	1.7323E-4	0.013858	0.013858	1.0
Valine, leucine and isoleucine biosynthesis	2/8	7.9884E-4	0.063109	0.021302	0.0
Phenylalanine metabolism	2/8	7.9884E-4	0.063109	0.021302	0.35714
Arginine and proline metabolism	2/36	0.016537	1.0	0.3237	0.14186
Valine, leucine and isoleucine degradation	2/40	0.020231	1.0	0.3237	0.0
Arginine biosynthesis	1/14	0.077404	1.0	0.99384	0.07614
Ubiquinone and other terpenoid-quinone biosynthesis	1/18	0.098517	1.0	0.99384	0.0
Pantothenate and CoA biosynthesis	1/20	0.10891	1.0	0.99384	0.0
Lipoic acid metabolism	1/28	0.14944	1.0	0.99384	0.0017
Glutathione metabolism	1/28	0.14944	1.0	0.99384	0.08873
Lysine degradation	1/30	0.15931	1.0	0.99384	0.0
Porphyrin metabolism	1/31	0.1642	1.0	0.99384	0.0
Glyoxylate and dicarboxylate metabolism	1/32	0.16908	1.0	0.99384	0.10582
Glycine, serine and threonine metabolism	1/33	0.17392	1.0	0.99384	0.25981
Fatty acid degradation	1/39	0.20248	1.0	1.0	0.0

Tyrosine metabolism	1/42	0.21642	1.0	1.0	0.13972

The table shows the detailed results from the pathway analysis. Since many pathways are tested at the same time, the statistical p values from enrichment analysis are further adjusted for multiple testings. Match status reflects hits/total, where total means the total number of compounds in the pathway, and the hits are the actually matched compounds from the list; the Raw p is the original p value calculated from the enrichment analysis; the Holm p is the adjusted p value calculated from the Holm-Bonferroni method; the FDR p is the p value adjusted using False Discovery Rate; the Impact is the pathway impact value calculated from pathway topology analysis. *FDR* false discovery rate.

Supplementary Table 3 | Results from the pathway analysis in maternal plasma/serum at >20 weeks' gestation or in the peripartum period.

Pathway name	Match status	Raw p	Holm adjusted p	FDR	Impact
Glyoxylate and dicarboxylate metabolism	2/32	0.003851	0.30808	0.30808	0.04233
Valine, leucine and isoleucine biosynthesis	1/8	0.025172	1.0	0.67057	0.0
Biotin metabolism	1/10	0.031385	1.0	0.67057	0.0
D-Amino acid metabolism	1/15	0.046779	1.0	0.67057	0.0
Butanoate metabolism	1/15	0.046779	1.0	0.67057	0.03175
Citrate cycle (TCA cycle)	1/20	0.061977	1.0	0.67057	0.04412
Pyruvate metabolism	1/23	0.071002	1.0	0.67057	0.0283
Alanine, aspartate and glutamate metabolism	1/28	0.085889	1.0	0.67057	0.08654
Lysine degradation	1/30	0.091791	1.0	0.67057	0.0
Sphingolipid metabolism	1/32	0.097661	1.0	0.67057	0.0
Cysteine and methionine metabolism	1/33	0.10059	1.0	0.67057	0.02184
Glycine, serine and threonine metabolism	1/33	0.10059	1.0	0.67057	0.21459
Arginine and proline metabolism	1/36	0.10931	1.0	0.67269	0.04767
Valine, leucine and isoleucine degradation	1/40	0.12084	1.0	0.69052	0.0

The table shows the detailed results from the pathway analysis. Since many pathways are tested at the same time, the statistical p values from enrichment analysis are further adjusted for multiple testings. Match status reflects hits/total, where total means the total number of compounds in the pathway, and the hits are the actually matched compounds from the list; the Raw p is the original p

value calculated from the enrichment analysis; the Holm p is the adjusted p value calculated from the Holm-Bonferroni method; the FDR p is the p value adjusted using False Discovery Rate; the Impact is the pathway impact value calculated from pathway topology analysis. *FDR* false discovery rate.

Supplementary Table 4 | Results from the pathway analysis in placental samples.

Pathway name	Match status	Raw p	Holm adjusted p	FDR	Impact
Ether lipid metabolism	1/20	0.037637	1.0	0.59607	0.0
Pyruvate metabolism	1/23	0.0432	1.0	0.59607	0.0
Glycolysis / Gluconeogenesis	1/26	0.048741	1.0	0.59607	0.0
Lipoic acid metabolism	1/28	0.052424	1.0	0.59607	0.0017
Glutathione metabolism	1/28	0.052424	1.0	0.59607	0.08873
Porphyrin metabolism	1/31	0.057929	1.0	0.59607	0.0
Glyoxylate and dicarboxylate metabolism	1/32	0.05976	1.0	0.59607	0.10582
Glycine, serine and threonine metabolism	1/33	0.061588	1.0	0.59607	0.25981
Glycerophospholipid metabolism	1/36	0.067058	1.0	0.59607	0.04814
Primary bile acid biosynthesis	1/46	0.085137	1.0	0.6811	0.00758

The table shows the detailed results from the pathway analysis. Since many pathways are tested at the same time, the statistical p values from enrichment analysis are further adjusted for multiple testings. Match status reflects hits/total, where total means the total number of compounds in the pathway, and the hits are the actually matched compounds from the list; the Raw p is the original p value calculated from the enrichment analysis; the Holm p is the adjusted p value calculated from the Holm-Bonferroni method; the FDR p is the p value adjusted using False Discovery Rate; the Impact is the pathway impact value calculated from pathway topology analysis. *FDR* false discovery rate.

Supplementary Figure 1 | Risk of bias for each included study. The risk of bias in each included study was assessed using a modified version of QUADOMICS, an adaptation of the Quality Assessment of Diagnostic Accuracy Studies (QUADAS) tool for studies using omic technologies. A total of eight domains were assessed. Each domain was judged as having a "low", "high", or "unclear" risk of bias. Source data are provided as a Source Data file.

Study	Selection of participants	Description of selection criteria	Description of procedures and timing of biological sample collection	Reporting of handling of specimens and pre-analytical procedures	Description of metabolite extraction methods and analytical techniques	Blinded interpretation of metabolomics results	Control for potential confounding variables	Avoiding of overfitting
Horgan 2010	?	−	+	+	+	?	+	−
Dessi 2011	?	−	?	+	+	?	+	−
Horgan 2011	+	?	+	+	+	+	+	?
Favretto 2012	+	+	?	?	+	?	+	−
Ivorra 2012	+	−	?	+	+	?	+	+
Bobiński 2013	+	?	?	+	+	?	−	−
Ryckman 2013	?	+	+	?	+	?	+	+
Sanz-Cortés 2013	+	+	+	+	+	?	+	?
Dessi 2014	?	−	?	+	+	?	−	?
Maitre 2014	+	?	?	?	+	?	+	?
Sulek 2014	+	?	+	+	+	?	?	−
Liu 2016	+	+	?	?	−	?	−	−
Abd El-Wahed 2017	+	+	−	−	−	?	?	−

**Visentin 2017**	+	+	+	?	+	?	–	–
**Delplancke 2018**	+	?	+	+	+	?	?	–
**Lu 2018**	+	?	?	?	+	?	+	–
**Miranda 2018**	+	+	+	+	+	?	+	?
**Bahado-Singh 2019**	+	?	+	+	+	?	+	?
**Alfano 2020**	+	+	+	+	+	?	+	?
**Briana 2020**	+	+	?	+	+	?	+	?
**Clinton 2020**	+	+	?	+	+	?	?	+
**Kan 2020**	+	–	+	+	?	?	–	–
**Sovio 2020**	+	+	+	+	+	?	+	+
**Welch 2020**	+	+	?	–	+	?	+	+
**Beken 2021**	+	+	+	?	+	?	–	–
**Byeon 2021**	+	?	?	+	+	?	?	–
**Morillon 2021**	+	?	+	+	+	?	+	+
**Moros 2021**	+	?	+	+	?	?	+	–
**Schupper 2021**	+	+	+	+	+	+	+	–
**Youssef 2021**	+	+	?	?	+	?	+	?
**Bahado-Singh 2022**	+	+	+	?	+	?	+	–

Chao de la Barca 2022	+	+	+	+	+	?	+	+
Gonzalez-Riano 2022	+	+	−	?	+	?	+	−
Karaer 2022	+	+	+	+	+	?	+	−
Liu 2022	+	+	?	?	?	?	+	−
McCarthy 2022	+	+	−	−	−	+	+	−
Umeda 2022	+	?	?	?	+	?	+	−
Voerman 2022	?	+	?	+	+	?	+	?
Bartho 2023	+	+	+	?	+	?	+	?
Chen 2023	+	+	+	?	?	?	+	?
Elhakeem 2023	+	+	−	?	+	?	+	+
Jafri 2023	+	+	?	?	−	?	−	−
Priante 2023	+	+	+	+	+	?	+	+
Tao 2023	+	+	+	+	+	?	?	−
Troisi, 2023	+	+	+	+	+	?	?	+
Yang 2023	+	+	+	+	+	?	+	−
Yeum 2023	+	+	−	−	+	?	+	+
Zhai 2023	+	+	+	+	+	?	+	?

+ Low risk of bias ? Unclear risk of bias − High risk of bias

Supplementary Figure 2 | Pathway analysis for significantly and consistently up- and down-regulated metabolites (N=5) that were reported in more than one study in maternal plasma/serum at >20 weeks' gestation or in the peripartum period. The metabolome view shows all matched pathways according to the p values from the pathway enrichment analysis and pathway impact values from the pathway topology analysis. Each circle in the figure represents a metabolic pathway. The colour of the circle indicates the significance level (Raw p) in the enrichment analysis; darker colour (redder) indicates greater significance. The size of the circle reflects the pathway impact value in the topology analysis, such that the larger the circle, the larger the impact value. There were no significantly enriched metabolic pathways (false discovery rate p value ≥0.05). FDR false discovery rate. Source data are provided as a Source Data file.

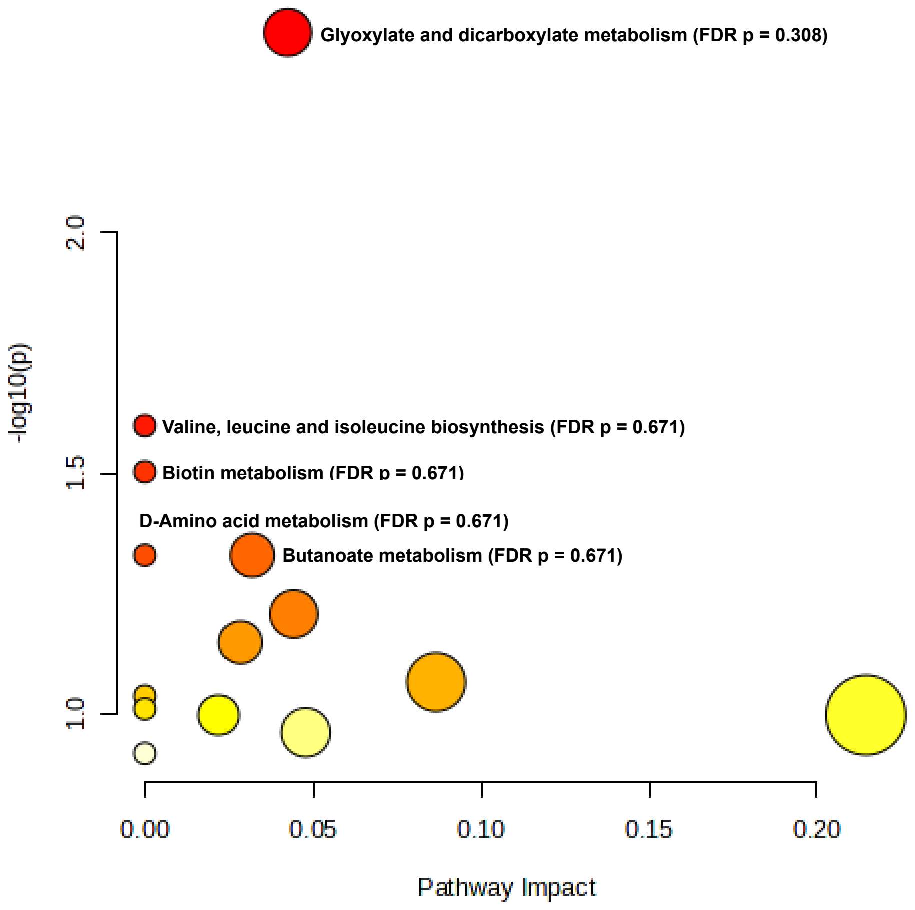

Supplementary Figure 3 | Pathway analysis for significantly and consistently up- and down-regulated metabolites (N=3) that were reported in more than one study in placental samples. The metabolome view shows all matched pathways according to the p values from the pathway enrichment analysis and pathway impact values from the pathway topology analysis. Each circle in the figure represents a metabolic pathway. The colour of the circle indicates the significance level (Raw p) in the enrichment analysis; darker colour (redder) indicates greater significance. The size of the circle reflects the pathway impact value in the topology analysis, such that the larger the circle, the larger the impact value. There were no significantly enriched metabolic pathways (false discovery rate p value ≥0.05). FDR false discovery rate. Source data are provided as a Source Data file.

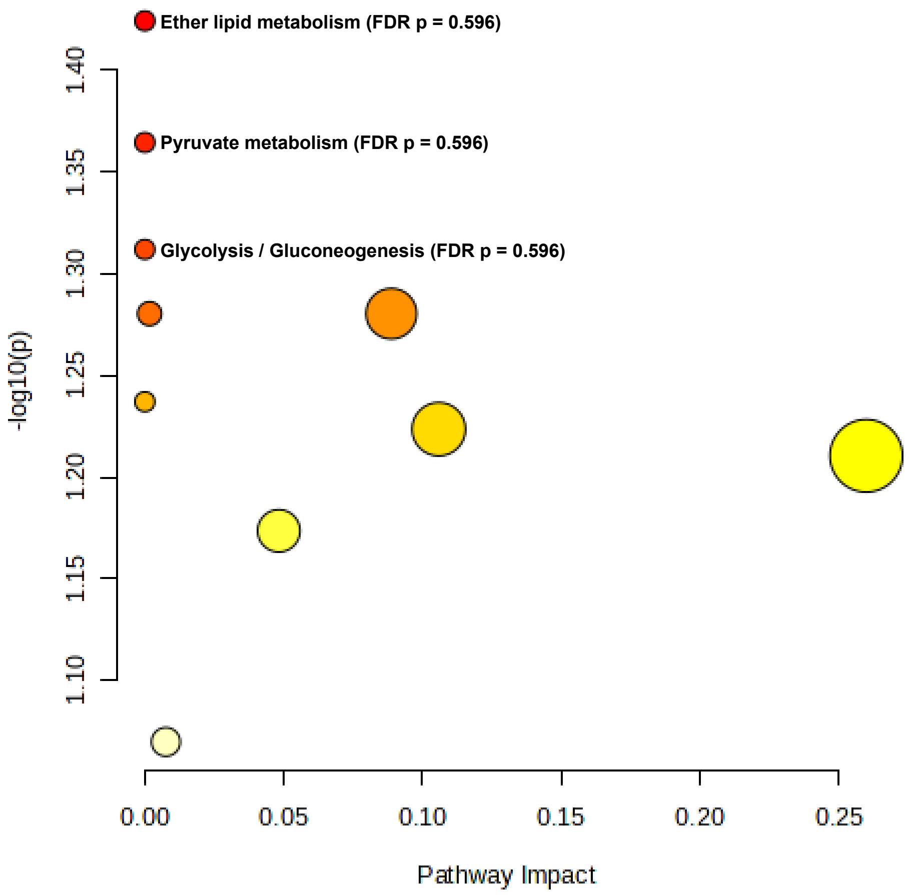

Supplementary Box 1

Metabolites significantly up-regulated or down-regulated in more than one study with a consistent direction of the association

Maternal plasma or serum at ≤20 weeks' gestation

Pregnanediol-3-glucuronide

Maternal plasma or serum at >20 weeks' gestation and in the peripartum period

Isoleucine
Lysine
Serine
4-aminobutyric acid
Malic acid

Maternal hair

Margaric acid
Myristic acid

Placenta

Glycine
Glycerophosphocholine
Lactic acid

Umbilical cord blood

LysoPC (16:1)
PC (36:3)
Leucine
Choline
Triglyceride
Glutamic acid
Trans-4-hydroxyproline
LysoPC (14:0)
LysoPC (16:0)
LysoPC (18:0)
LysoPC (20:4)
PC (36:1)
PC (36:4)
PC (38:4)
PC (40:4)
Decanoyl carnitine
Dodecanoic acid
2-aminoadipic acid
Stearic acid
Gamma-linolenic acid

Eicosatrienoic acid
Arachidonic acid
Cholesterol HDL
Glucose

Newborn dried blood spot

Free carnitine
Butyryl carnitine
Acetyl carnitine
Decenoyl carnitine
Propionyl carnitine
Proline
Phenylalanine
Leucine
Glycine
Tyrosine
Valine
Arginine
Octadecadienyl carnitine
Isovaleryl carnitine
Tetradecanoyl carnitine
Dodecanoyl carnitine
Octadecanoyl carnitine
Octadecenoyl carnitine

Newborn urine

Myo-inositol
Creatinine
Creatine
