# Supplementary material for: Metabolomic signatures associated with fetal growth restriction and small for gestational age: a systematic review
Source: Nat Commun. 2024 Nov 11;15:9752. doi: 10.1038/s41467-024-53597-4 (PMC11555221; doi:10.1038/s41467-024-53597-4)
Supplement: Supplementary file 2 — Reporting Summary [file 41467_2024_53597_MOESM2_ESM.pdf]

nature portfolio

Corresponding author(s): Agustin Conde Agudelo and Jose Villar

Last updated by author(s): Sep 24, 2024

Reporting Summary

Nature Portfolio wishes to improve the reproducibility of the work that we publish. This form provides structure for consistency and transparency in reporting. For further information on Nature Portfolio policies, see our Editorial Policies and the Editorial Policy Checklist.

Statistics

For all statistical analyses, confirm that the following items are present in the figure legend, table legend, main text, or Methods section.

n/a	Confirmed	
☐	☒	The exact sample size (*n*) for each experimental group/condition, given as a discrete number and unit of measurement
☒	☐	A statement on whether measurements were taken from distinct samples or whether the same sample was measured repeatedly
☐	☒	The statistical test(s) used AND whether they are one- or two-sided<br>*Only common tests should be described solely by name; describe more complex techniques in the Methods section.*
☒	☐	A description of all covariates tested
☐	☒	A description of any assumptions or corrections, such as tests of normality and adjustment for multiple comparisons
☐	☒	A full description of the statistical parameters including central tendency (e.g. means) or other basic estimates (e.g. regression coefficient) AND variation (e.g. standard deviation) or associated estimates of uncertainty (e.g. confidence intervals)
☐	☒	For null hypothesis testing, the test statistic (e.g. *F*, *t*, *r*) with confidence intervals, effect sizes, degrees of freedom and *P* value noted<br>*Give P values as exact values whenever suitable.*
☒	☐	For Bayesian analysis, information on the choice of priors and Markov chain Monte Carlo settings
☒	☐	For hierarchical and complex designs, identification of the appropriate level for tests and full reporting of outcomes
☒	☐	Estimates of effect sizes (e.g. Cohen's *d*, Pearson's *r*), indicating how they were calculated

Our web collection on statistics for biologists contains articles on many of the points above.

Software and code

Policy information about availability of computer code

Data collection	No primary data collection was carried out for this analysis. Data was collected manually from published studies.
Data analysis	We used MetaboAnalyst 5.0 online software (https://www.metaboanalyst.ca) for pathway analysis.

For manuscripts utilizing custom algorithms or software that are central to the research but not yet described in published literature, software must be made available to editors and reviewers. We strongly encourage code deposition in a community repository (e.g. GitHub). See the Nature Portfolio guidelines for submitting code & software for further information.

Data

Policy information about availability of data

All manuscripts must include a data availability statement. This statement should provide the following information, where applicable:

- Accession codes, unique identifiers, or web links for publicly available datasets
- A description of any restrictions on data availability
- For clinical datasets or third party data, please ensure that the statement adheres to our policy

Data that support the findings of this study have been deposited in the Mendeley Data Repository: https://data.mendeley.com/datasets/4rsm8zv5x3/1. Source data are provided with this paper.

Research involving human participants, their data, or biological material

Policy information about studies with human participants or human data. See also policy information about sex, gender (identity/presentation), and sexual orientation and race, ethnicity and racism.

Reporting on sex and gender	Primary data collection was not carried out for this systematic review, so the study does not involve human research participants. As stated in the Methods section, our analyses are specific to pregnant women and newborns (both men and women). We included all available data regardless of whether the input study collected and reported data by sex and gender of newborns.
Reporting on race, ethnicity, or other socially relevant groupings	Primary data collection was not carried out for this systematic review, so the study does not involve human research participants. We included all available data regardless of whether the input study collected and reported data by race or ethnicity of pregnant women or newborns.
Population characteristics	Primary data collection was not carried out for this systematic review. The study aimed to identify metabolomic signatures in biological samples of pregnant women and/or newborns to understand the pathophysiology of fetal growth restriction and/or small for gestational age.
Recruitment	Primary data collection was not carried out for this systematic review, so we did not recruit participants.
Ethics oversight	This systematic review did not need ethics committee or institutional review board approval because all included studies were published previously and had each previously received local institutional review board approvals and written consent from participants.

Note that full information on the approval of the study protocol must also be provided in the manuscript.

Field-specific reporting

Please select the one below that is the best fit for your research. If you are not sure, read the appropriate sections before making your selection.

☒ Life sciences ☐ Behavioural & social sciences ☐ Ecological, evolutionary & environmental sciences

For a reference copy of the document with all sections, see nature.com/documents/nr-reporting-summary-flat.pdf

Life sciences study design

All studies must disclose on these points even when the disclosure is negative.

Sample size	No sample size calculation was performed for this systematic review. All available studies meeting inclusion criteria were included regardless sample size. As reported in the main text Results section, 48 studies, which included a total of 4228 women and 820,271 newborns, met the inclusion criteria.
Data exclusions	As described in the Methods section, studies not using a metabolomics technology, animal and in vitro studies, studies including multiple pregnancies or neonates that did not report singleton data separately, conference abstracts, case reports, letters, editorials, and reviews were excluded from the systematic review.
Replication	This is a systematic review of existing studies which are publicly available. Therefore, the analyses can be replicated.
Randomization	This analysis is a systematic review of existing studies and thus, there were no experimental groups.
Blinding	Blinding was not relevant to this study, as it was a systematic review of existing data and we did not collect primary data.

Reporting for specific materials, systems and methods

We require information from authors about some types of materials, experimental systems and methods used in many studies. Here, indicate whether each material, system or method listed is relevant to your study. If you are not sure if a list item applies to your research, read the appropriate section before selecting a response.

Materials & experimental systems

n/a	Involved in the study
☒	☐ Antibodies
☒	☐ Eukaryotic cell lines
☒	☐ Palaeontology and archaeology
☒	☐ Animals and other organisms
☒	☐ Clinical data
☒	☐ Dual use research of concern
☒	☐ Plants

Methods

n/a	Involved in the study
☒	☐ ChIP-seq
☒	☐ Flow cytometry
☒	☐ MRI-based neuroimaging

Plants

Seed stocks	NA
Novel plant genotypes	NA
Authentication	NA
